# System design for monitoring REDOX potential in treated water using an ORP sensor

**DOI:** 10.1016/j.ohx.2025.e00717

**Published:** 2025-10-19

**Authors:** A. Escobar-Díaz, A. Villarreal, B. Zárate-Nicolas, A. Rojas-Olivos

**Affiliations:** a Escuela de Ingenierias y Arquitectura, Universidad La Salle Oaxaca A.C., Mexico; b Facultad de Ciencias, Universidad Nacional Autónoma de México (UNAM), Ciudad de México, Mexico; c Centro Interdisciplinario de Investigación para el Desarrollo Integral Regional (CIIDIR), Unidad Oaxaca, Instituto Politécnico Nacional (IPN), Oaxaca, Mexico; d SECIHTI-Instituto Politécnico Nacional, CIIDIR Unidad Oaxaca, Hornos 1003, Santa Cruz Xoxocotlán, Oaxaca, Z.C. 71230, Mexico

**Keywords:** Water treatment, Antimicrobial measurement, Data acquisition system, Water sanitation, Environmental sensor

## Abstract

Electrodes that measure the oxidation–reduction potential (ORP) assess the relationship between oxidized and reduced substances that may exist in water disinfection processes. This work presents the implementation and characterization of an ORP measurement system using the Atlas Scientific ORP sensor, integrated with an Arduino R4 development board as the control and data transmission unit. The main objective is to develop an economical, fast, and reliable solution for the continuous monitoring of the bacteriological quality of water in a wastewater treatment plant (WWTP). The system operates in real time, transmitting information to remote platforms. The manufacturing and integration stages of the system, as well as its calibration and experimental validation, are described. The results indicate that a properly calibrated sensor provides accurate measurements, making it a viable and cost-effective alternative to traditional laboratory equipment or bacteriological analyses that require long processing times. Additionally, by monitoring the ORP variable in a WWTP, alerts can be generated for variations related to microbial load in the wastewater treatment process, enabling immediate corrective actions before discharge into surface water bodies.

## Graphical abstract

**Figure:**
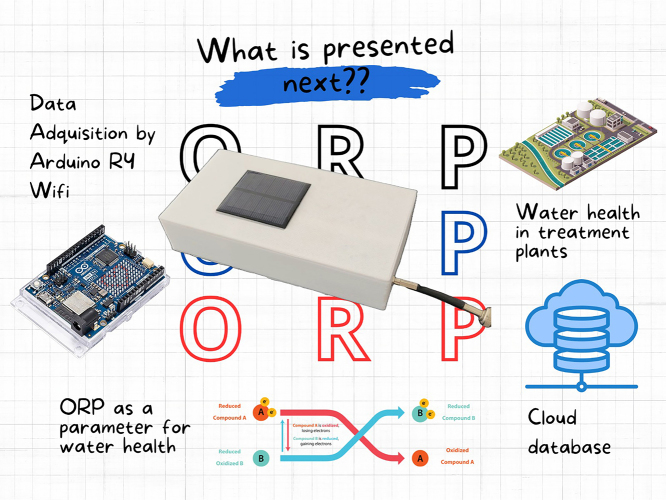

## Specifications table

**Table:** 

Hardware name	SmartORP

Subject area	•Environmental, Planetary and Agricultural Sciences

Hardware type	•Field measurements and sensors

Commercial analog	•Hanna Instruments HI98121 (portable pH/ORP meter)

Open source license	The designed hardware, including the probe housing and holder, is released under the CERN Open Hardware License v2-S. Additional software developed for network communication and cloud storage is released under the MIT License. Commercial components are used with their proprietary software, so the ORP sensor control code is not included in the open source license.

Cost of hardware	120 Usd

Source file repository	https://doi.org/10.5281/zenodo.14983410

## 1 Hardware in context

Water quality monitoring is a very important aspect of wastewater treatment plant operation, typically performed via parameters such as oxidation–reduction potential (ORP). Ensuring an efficient treatment process and compliance with environmental regulations depends on continuously tracking these parameters [1]. Monitoring the bacteriological quality of water likewise plays a crucial role in wastewater treatment plant management. However, many communities face high operating costs, including the cost of testing fecal coliforms in certified laboratories. These analyses – requiring sampling, transport, and 48–72 h of turnaround – prevent real-time tracking of parameters associated with microbial load. Among potential real-time indicators, ORP is especially valuable because it provides insight into the disinfection process [2]. According to Mexican regulations and the World Health Organization (WHO), values above 650 mV generally indicate the absence of bacteriological contamination by Escherichia coli, whereas values around 450 mV or lower suggest that adequate disinfection has not been achieved [3], [4]. On the other hand, remote monitoring systems make continuous data acquisition possible, offering an essential tool for detecting rapid changes in water quality—such as high organic loads at the process inlet or mechanical issues in critical stages [5], [6]. By delivering real-time data without a constant onsite presence, remote monitoring reduces operating costs while minimizing disruption to local ecosystems [7]. The use of IoT-enabled sensors further integrates data into cloud platforms, allowing remote access, collaborative analysis, and more efficient resource management for municipal authorities, researchers, and communities [8], [9], [10]. In evaluating the reliability of monitoring methods, Bland-Altman plots and linear regression provide statistical insights that help verify measurement accuracy and identify sources of bias [11]. Bland-Altman plots show the mean differences and limits of agreement, while linear regression models relationships between variables—valuable for predicting future outcomes based on historical data [12], [13]. Additionally, multivariate analyses can uncover complex patterns in water quality data, leading to improved operational strategies in diverse contexts [14]. By incorporating these statistical tools, an ORP monitoring system not only enhances the reliability of each individual measurement but also facilitates long-term trend analysis of bacteriological water quality—vital for sustainable wastewater treatment planning and timely decisions about corrective or preventive measures [15], [16].

To facilitate the reading of this work, Table 1 summarizes the acronyms and symbols used throughout the manuscript. This helps ensure consistency and provides quick reference for readers who are not familiar with specific terminology.

**Table 1:** List of acronyms and symbols used in the manuscript.

Acronym/Symbol	Meaning
ORP	Oxidation-Reduction Potential (Redox Potential)
HI9829	Multiparameter reference system (Hanna Instruments)
IoT	Internet of Things
CCC	Concordance Correlation Coefficient
SD	Standard Deviation
CI	Confidence Interval
ADC	Analog-to-Digital Converter
mV	Millivolt
PCB	Printed Circuit Board
Wi-Fi	Wireless Fidelity

## 2 Hardware description

The ORP measurement system developed in this work integrates the Gravity ORP Meter V2.0 electrochemical sensor from Atlas Scientific with an Arduino R4 development board. This configuration emphasizes modularity, low cost, and wireless real-time data transmission. A platinum electrode is used to capture redox measurements from the physical environment, and the system is powered by a rechargeable battery connected to a solar cell, providing autonomous operation in field conditions.

Unlike traditional ORP monitoring systems that rely on bulky and expensive laboratory equipment, this solution is designed for portable and continuous in-situ deployment. The integration with the Arduino platform enables seamless connection to Internet of Things (IoT) services, allowing data to be stored and analyzed in the cloud. This expands its usability for long-term monitoring and large-scale deployments.

Customization is a key feature of this system. The firmware can be adapted to different transmission protocols and platforms, while the physical design – including the sensor holder and protective enclosure – can be modified and reprinted for various contexts. All mechanical components are released under the CERN Open Hardware License v2-S, and the communication software is released under the MIT License, ensuring open-source reusability and adaptability. The sensor’s proprietary control software, however, is not included.

Compared to pre-existing methods, the proposed system stands out for its affordability, ease of implementation, and adaptability. Its low cost makes it accessible for low-resource settings, and the plug-and-play nature of the Arduino platform allows for rapid deployment without technical expertise. Its wireless connectivity facilitates remote data collection, enabling early detection of anomalies in water quality.

• Enables low-cost, real-time ORP monitoring in remote or off-grid locations with solar-powered operation.
• Serves as a practical educational tool for teaching environmental sensing and electrochemical measurements.
• Can be expanded with additional sensors (pH, conductivity, temperature) to build custom water quality stations.
• Facilitates research on advanced oxidation and disinfection processes through continuous field measurements.
• Offers a flexible open-source platform for integration into IoT networks and development of predictive models.

## 3 Design files summary

**Table:** 

Design filename	File type	Open source license	Location of the file
Case_proyect_ORP	SolidWorks files	Creative Commons for Open Source Hardware (COSH)	Link
Tapa_proyect_ORP	SolidWorks files	Creative Commons for Open Source Hardware (COSH)	Link
Pieza_Holder	SolidWorks files	Creative Commons for Open Source Hardware (COSH)	Link

**Case (Case_proyect_ORP)** This design features three individual compartments, each separated by 2-mm-thick walls. The walls include openings that connect all three sections, positioned at a specific height to ensure that if one compartment floods, it will not affect the others.

**Cover (Tapa_proyect_ORP)** Designed as a complement to the case, this cover snaps into place and remains flush with the outer walls of the enclosure. It includes a space to hold the solar cell that powers the system’s battery, as well as an opening that enables communication or cable routing as needed.

**Holder (Pieza_Holder)** This part is designed to channel water into the internal reservoir in a way that prevents a strong current from forming. It is installed at the treatment plant’s outflow and also provides a mount for the ORP electrode.

## 4 Bill of materials

**Table:** 

Designator	Component	Number	Cost per unit	Total cost	Source of materials	Material type
Data-processing	Arduino R4 development board	1	30.00 USD	30.00 USD	Amazon	Other
ORP-Sensor1	ORP sensor	1	24.99 USD	24.99 USD	Atlas	Other
ORP-Physical-Sense2	ORP electrode	1	36.34 USD	36.34 USD	MercadoLibre	Other
Case-proyect-ORP1	PETG filament	0.1	18.51 USD/kg	1.85 USD	MercadoLibre	Polymer
charging-system 1	Battery	2	3.23 USD	6.46 USD	MercadoLibre	Metal/Inorganic
charging-system 2	Solar cell	1	4.84 USD	4.84 USD	Steren	Other
Charger	Solar panel controller	1	1.03 USD	1.03 USD	ebay	Other

In order to highlight the practical advantages of the proposed system, Table 2 compares its main specifications, cost, and features with representative commercial ORP monitoring devices. This comparison emphasizes the affordability and deployability of the hardware in low-resource environments.

**Table 2:** Comparison of the developed system with commercial alternatives.

Device	Measurement range (mV)	Accuracy	Cost (USD)
**This work (Arduino R4 + ORP Sensor)**	−1500–1500	±1 mV	∼120
Hanna HI9829	−2000–2000	±0.5 mV	∼5400
Hanna HI98120	−2000–2000	±1 mV	∼250
Vernier ORP sensor^a^	−2000–2000	±2 mV	∼170

a Sensor only, without additional electronics.

## 5 Build instructions

Building and integrating the ORP measurement system with the Arduino R4 board involves several steps, from connecting the components to the final configuration for sending data to the cloud.

### 5.1 Electrical connection and assembly

The system must be assembled in an orderly manner, ensuring that all connections are secure and protected from environmental exposure.

#### 5.1.1 Connecting the ORP sensor to the Arduino R4 board

• Identify the sensor’s analog output pin (usually marked “OUT” or “A”).
• Connect GND to the Arduino R4 GND.
• Connect VCC to the 5 V line of the Arduino R4 (as specified in the sensor datasheet).
• Connect the analog output to analog pin A0 of the Arduino R4.

#### 5.1.2 Power system connection (Battery, Controller, and Solar cell)

• Connect the solar cell to the charge controller: connect the positive terminal of the solar cell to the “S+” pin on the controller, and the negative terminal to the “S–” pin.
• Connect the battery to the charge controller: connect the positive terminal of the battery to the “B+” pin on the controller, and the negative terminal to the “B–” pin.
• Connect the regulated output from the controller to the Arduino R4 power input: connect the Arduino’s “$V_{in}$” pin to the “L+” pin on the controller’s output, and the Arduino’s “GND” pin to the “L–” pin.

### 5.2 Protection and mounting of the housing

**Fig. 1:**
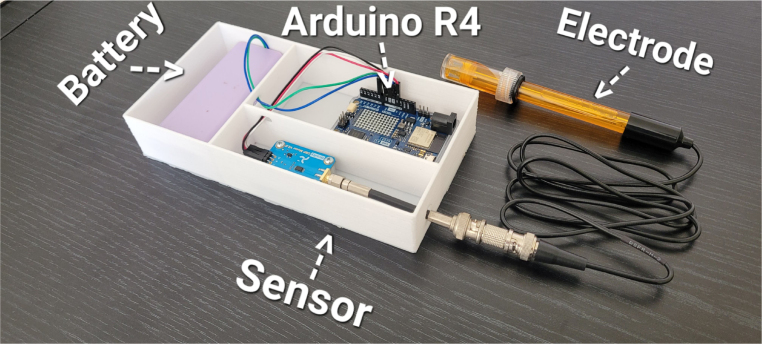
SolidWorks-designed enclosure with internal component layout.
• Seal the joints using silicone or rubber gaskets to prevent moisture and dust ingress.
• Place desiccants inside to reduce internal humidity.
• Mount the solar panel in a well-lit location, oriented for optimal charging (Fig. 2).

Since the device will be exposed to the environment, the enclosure (see Case_proyect_ORP) must protect the internal components:

• Insert the Arduino and battery into the enclosure (see Fig. 1).

**Fig. 2:**
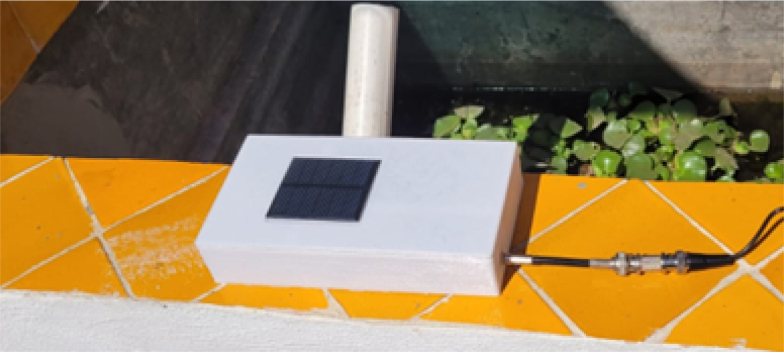
Solar cell mounted in a well-lit location.

### 5.3 Correct installation of the sensor in the test medium

**Fig. 3:**
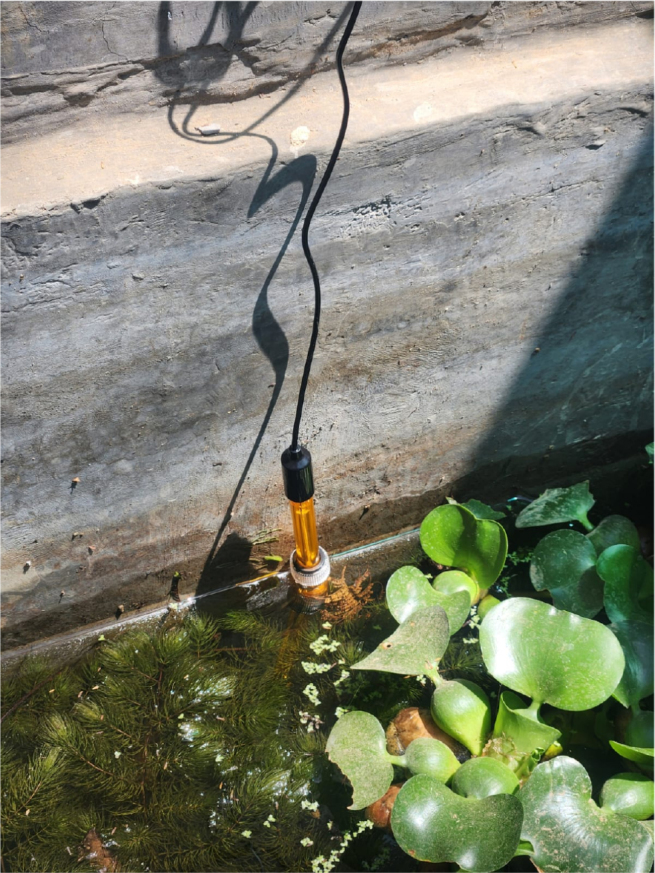
Electrode position submerged in water.
• Secure the electrode to avoid movement or detachment.

To ensure accurate readings, install the electrode (see Pieza_Holder) correctly:

• Use a PVC-T connection when mounting in a pipe for stable flow.
• Avoid locations with high turbulence or very fast flow.
• In tanks, immerse the electrode 3 cm below the waterline (Fig. 3).

### 5.4 Firmware and connectivity settings

Once assembled, program the Arduino R4 using the Arduino IDE:

• Select the appropriate board and COM port.
• Load code to:
  – Read analog values from A0.
  – Apply the voltage-to-ORP conversion.
  – Establish Wi-Fi connection with user credentials.
  – Transmit data to ArduinoCloud or a selected cloud service.
  – Define alerts using three-point thresholds for anomaly detection.
• Calibrate the sensor using a 225 mV buffer and the command: CAL,(225).

### 5.5 System verification and cloud monitoring

• Power the device and verify the sensor is working.
• Access the monitoring platform and check real-time updates.
• Create dashboards and alerts.
• Use platforms such as Google Sites for public access if needed.

### 5.6 Limitations

• **Single input (SMA to BNC):** Only one sensor can be connected per board without additional adapters.
• **Calibration required:** Must be done every 3–4 weeks for accuracy.
• **No heat dissipation:** The case lacks cooling features; avoid prolonged sun exposure to prevent overheating.

## 6 Operation instructions

Proper operation of the system ensures reliable measurements from the ORP monitoring system for continuous use. The following instructions combine general recommendations with specific actions.

### Startup and monitoring values

• Power on the system and ensure the ORP sensor is fully submerged and free of air bubbles at the tip.
• Wait for a stabilization period (typically a few seconds) for the ORP values to appear on the selected platform (e.g., ArduinoCloud).
• Open the visualization dashboard (ArduinoCloud or Google Sites) to monitor the ORP readings in real time.

During operation, fluctuations may occur due to variations in water chemistry or external disturbances (e.g., turbulence or stray currents). Regular verification using calibration buffers or reference instruments is recommended.

### Calibration and verification of readings

• Calibrate the system using a standard ORP buffer solution (typically 225 mV) when needed.
• If measurement drift is detected, adjust the conversion formula in the code or apply an offset correction.
• Perform calibration and adjustments in a clean environment to avoid contamination.

Connectivity is essential for continuous monitoring. Ensure a stable Wi-Fi connection. If operating in industrial areas with high electrical noise, consider:

• Using a GSM module as backup communication.
• Implementing analog or digital filters to reduce signal noise.
• Avoiding equipment that generates strong electromagnetic fields near the sensor.

### Maintenance and safety

• **Initial pretreatment:** Before first use, immerse the ORP electrode in a standard oxidizing or reducing solution depending on the target application.
• **Routine cleaning:** Clean the electrode regularly, especially if scale or deposits form. Use deionized water and mild cleaning agents (e.g., diluted hypochlorite or 5%–10% HCl).
• **Specialized cleaning:** If needed, use manufacturer-recommended cleaning solutions such as 0.034% hydrochloric acid.
• **After cleaning:** Dry the electrode completely and store it in ORP electrode storage solution in the cap.
• **Long-term storage:** Clean the probe after extended periods of inactivity due to possible crystallization of the storage solution. Recalibration is required afterward.
• **Protective gear:** Wear gloves and safety glasses when handling chemicals or the sensor.
• **Safe handling:** Disconnect power before handling wiring or removing the sensor. Avoid contact with metal surfaces.
• **Use limitations:** Do not expose the electrode continuously to strong oxidizers or reducers to prevent premature degradation.
• **Mechanical protection:** Avoid impacts or stress on the sensor housing or wiring.

These minimum recommendations help ensure long-term reliability of the system and improve measurement accuracy. Following them enables stable real-time transmission, early anomaly detection, and dependable data collection across diverse environments.

## 7 Validation and characterization

To evaluate the performance of the proposed system (*ORP_A*), various analytical techniques were implemented to characterize its accuracy, agreement, and sensitivity compared to a reference sensor (Hanna HI9829-10041 Multiparameter ORP-H29). These techniques include linear regression analysis, Deming regression, sensitivity analysis, the Bland-Altman plot, and the concordance correlation coefficient (CCC). These methodologies help determine the relationship between sensors, identify potential biases, and assess the system’s responsiveness under different redox conditions.

For testing, dilutions of potassium permanganate (KMnO_4_) and sodium thiosulfate (Na_2_S_2_O_3_) were used to generate a range of oxidation–reduction potentials (ORP), covering high and low redox states to evaluate sensor behavior.

### 7.1 Linear regression analysis

Linear regression analysis was used to evaluate the relationship between the ORP_A system and the reference sensor (HI9829). The objective was to determine whether the values measured by ORP_A correlate linearly with those from the Ref. [17].

**Fig. 4:**
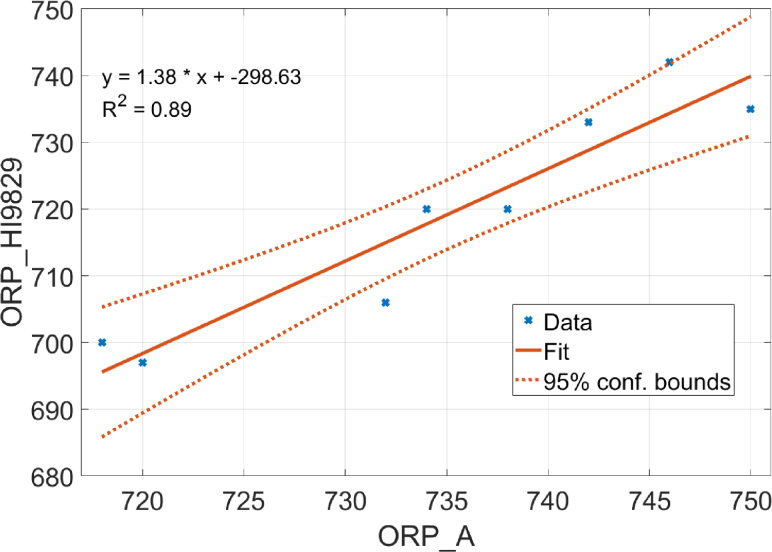
Linear regression curve between the Atlas sensor and the reference system.

The resulting regression equation was:

(1) $${\text{ORP}}_{HI9829}\;\left[\text{mV}\right]=1.38\cdot {\text{ORP}}_{A}\;\left[\text{mV}\right]-298.63\;\left[\text{mV}\right]$$

with a determination coefficient $R^{2}=0.89$, indicating a strong linear correlation. This result suggests a systematic shift in scale, but overall consistency between measurements (see Fig. 4).

### 7.2 Deming regression

Deming regression accounts for measurement errors in both axes [18]. The regression equation was:

(2) $${\text{ORP}}_{HI9829}\;\left(\text{mV}\right)=1.04\cdot {\text{ORP}}_{A}\;\left(\text{mV}\right)-44.26\;\left(\text{mV}\right)$$

**Fig. 5:**
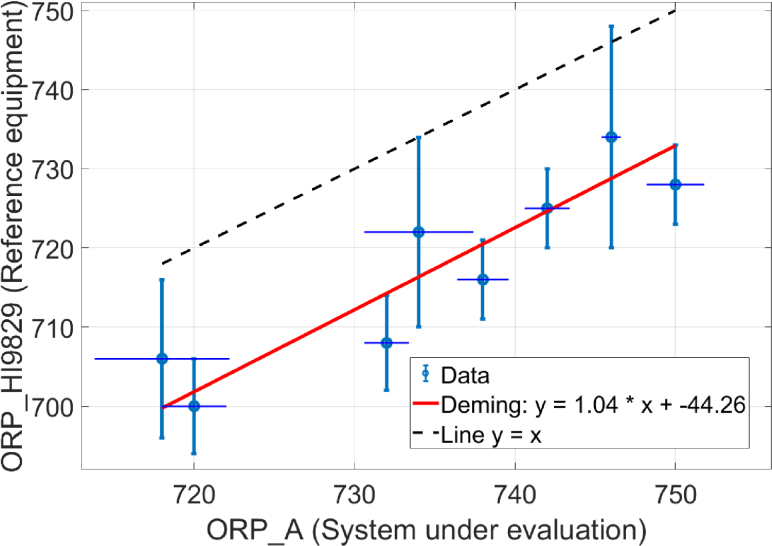
Deming regression for system comparison.

The variance ratio (λ) was calculated as:

(3) $$\lambda =\frac{\sigma_{{\text{ORP}}_{A}}^{2}\;\left({\text{mV}}^{2}\right)}{\sigma_{{\text{ORP}}_{HI9829}}^{2}\;\left({\text{mV}}^{2}\right)}$$

with λ=1.0667, indicating slightly higher variability in the ORP_A system. The slope is closer to 1 than in linear regression, suggesting less systematic bias when accounting for measurement uncertainty (see Fig. 5)

### 7.3 System sensitivity

**Fig. 6:**
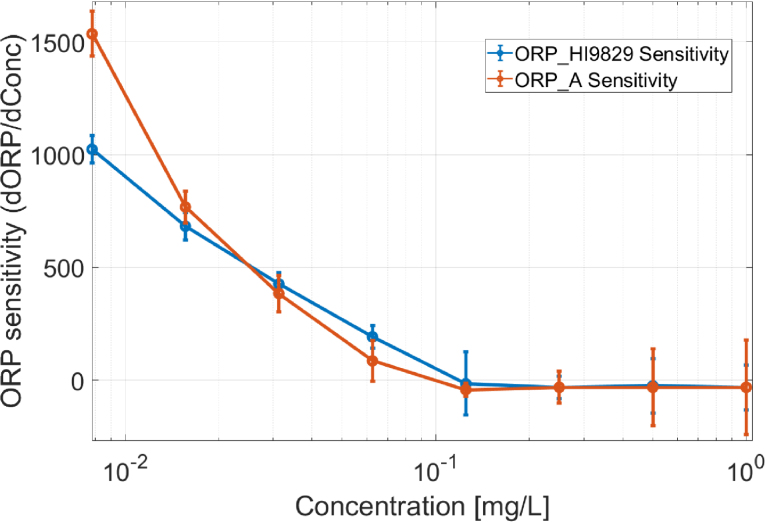
ORP system sensitivity vs. concentration.

System sensitivity (S) was calculated using:

(4) $$S\;\left(\text{mV}\;\text{L}/\text{mg}\right)=\frac{\Delta \text{ORP}\;\left(\text{mV}\right)}{\Delta C\;\left(\text{mg}/\text{L}\right)}$$

where ΔORP is the change in measured potential (mV) and ΔC is the change in concentration of redox species (mg/L). Both ORP_A (the proposed device) and the commercial reference (HI9829) showed decreasing sensitivity at higher concentrations, though their response was consistent across the tested range (see Fig. 6)

### 7.4 Bland-Altman analysis

This method examines agreement by plotting the difference vs. the average of two measurements [19]:

(5) $$\text{Difference}\;\left(\text{mV}\right)={\text{ORP}}_{HI9829}\;\left(\text{mV}\right)-{\text{ORP}}_{A}\;\left(\text{mV}\right)$$

The limits of agreement are calculated as:

(6) μ(mV)±1.96⋅σ(mV)

where μ is the mean of the differences and σ is their standard deviation.

**Fig. 7:**
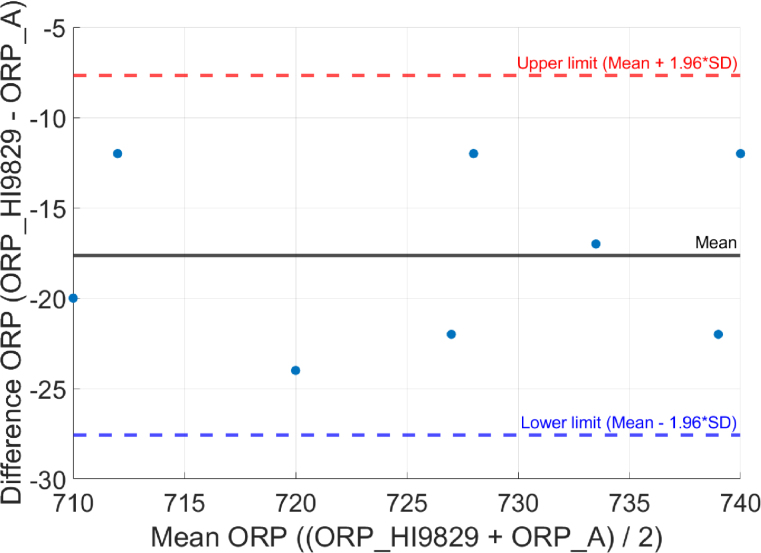
Bland-Altman curve comparing ORP_HI9829 and ORP_A.

In this analysis, a mean error of −17.62 mV and standard deviation of 5.07 mV were obtained. All points fell within the limits of agreement, confirming consistent and predictable performance of the system (see Fig. 7)

### 7.5 Concordance Correlation Coefficient (CCC)

The concordance correlation coefficient (CCC) evaluates both accuracy and precision, and is calculated as:

(7) $$CCC=\frac{2R\cdot \sigma_{x}\cdot \sigma_{y}}{\sigma_{x}^{2}+\sigma_{y}^{2}+{\left(\mu_{x}-\mu_{y}\right)}^{2}}$$

where $\sigma_{x}$ and $\sigma_{y}$ are the standard deviations (mV), $\mu_{x}$ and $\mu_{y}$ are the mean values (mV) of ORP_A and ORP_HI9829, and R is Pearson’s correlation coefficient [20].

A CCC value of 0.4247 indicates moderate agreement between the sensors, considering both systematic and random error sources.

The ORP_A system shows strong linear correlation with the reference sensor, minor systematic underestimation (correctable by calibration), and consistent sensitivity and agreement across redox concentrations. These findings support its use as a cost-effective and reliable alternative for ORP monitoring in real-world environments.

## CRediT authorship contribution statement

**A. Escobar-Díaz:** Writing – original draft, Methodology, Data curation. **A. Villarreal:** Writing – review & editing, Writing – original draft, Methodology, Investigation, Data curation. **B. Zárate-Nicolas:** Writing – review & editing, Resources, Methodology. **A. Rojas-Olivos:** Writing – review & editing, Writing – original draft, Methodology, Conceptualization.

## Declaration of competing interest

The authors declare the following financial interests/personal relationships which may be considered as potential competing interests: Alejandra Rojas-Olivos reports financial support was provided by National Council for Science and Technology. If there are other authors, they declare that they have no known competing financial interests or personal relationships that could have appeared to influence the work reported in this paper.
